# Real‐World Investigation of Satralizumab in Patients With Neuromyelitis Optica Spectrum Disease

**DOI:** 10.1002/acn3.70246

**Published:** 2025-11-14

**Authors:** Li‐Tsung Lin, Hui‐An Lin, Sheng‐Feng Lin

**Affiliations:** ^1^ Department of Ophthalmology Chang Gung Memorial Hospital, Linkou Medical Center Taoyuan Taiwan; ^2^ Department of Emergency Medicine School of Medicine, College of Medicine, Taipei Medical University Taipei Taiwan; ^3^ Department of Emergency Medicine Taipei Medical University Hospital Taipei Taiwan; ^4^ Department of Public Health, School of Medicine College of Medicine, Taipei Medical University Taipei Taiwan; ^5^ School of Public Health College of Public Health, Taipei Medical University Taipei Taiwan; ^6^ Department of Evidence‐Based Center Taipei Medical University Hospital Taipei Taiwan; ^7^ Department of Medical Research Taipei Medical University Hospital Taipei Taiwan

**Keywords:** interleukin‐6 (IL‐6), neuromyelitis optica spectrum disease (NMOSD), satralizumab

## Abstract

**Objective:**

Satralizumab, a monoclonal antibody targeting the interleukin‐6 receptor, has demonstrated efficacy in clinical trials for neuromyelitis optica spectrum disorder (NMOSD). However, its real‐world effectiveness and safety compared to conventional immunosuppressive therapies remain uncertain.

**Methods:**

We identified patients diagnosed with NMOSD in the TriNetX federated health research platform between January 2018 and April 2024. This international platform was accessed via Taipei Medical University. Patients were followed for 1 month to 3 years. A 1:1 propensity‐score matching (PSM) analysis balanced baseline characteristics between the satralizumab and conventional immunosuppressant groups. Risk ratios (RRs) were calculated for relapse risk and safety outcomes, including sepsis, respiratory infection, urinary tract infection, anemia, neutropenia, and mortality.

**Results:**

A total of 220 patients received satralizumab, while 1744 received conventional immunosuppressants. After PSM, 218 patients remained in each group. Satralizumab was associated with a significantly lower relapse risk at 1 month (RR: 0.38, 95% CI 0.21–0.66), 3 months (RR: 0.43, 95% CI 0.28–0.66), 6 months (RR: 0.50, 95% CI 0.33–0.70), 9 months (RR: 0.62, 95% CI 0.46–0.83), 12 months (RR: 0.63, 95% CI 0.47–0.83), 24 months (RR: 0.60, 95% CI 0.42–0.86) and 36 months (RR: 0.52, 95% CI 0.32–0.83). Across all follow‐up intervals, numbers needed to treat were consistently between 4 and 9. No significant differences were observed in infection rates, anemia, neutropenia, or mortality between the groups.

**Interpretation:**

Satralizumab demonstrated superior efficacy in reducing NMOSD relapse rates compared to conventional immunosuppressants while maintaining a comparable safety profile.

## 1 Introduction

Neuromyelitis optica spectrum disorder (NMOSD) is a severe autoimmune inflammatory disorder of the central nervous system characterized by recurrent attacks that primarily affect the optic nerves and spinal cord [1, 2]. The hallmark clinical presentation of NMOSD includes severe optic neuritis, which is often bilateral, and longitudinally transverse myelitis [3]. Inflammation can also extend to other regions of the central neural system, such as the brainstem, causing oculomotor dysfunction, diplopia, and nystagmus. Involvement of the area postrema frequently manifests as intractable nausea, vomiting, and hiccups, sometimes mimicking gastroenteritis or cyclical vomiting syndrome.

NMOSD has a relapsing course up to 97.5% of patients [4], leading to progressive neurological disability. A single relapse can significantly impact a patient's quality of life, underscoring the urgent need for effective relapse prevention in NMOSD management [5]. Without treatment, approximately 50% of patients may become wheelchair‐bound or blind, and around 30% may die within 5 years of the first attack [2]. Therefore, immunosuppressive therapies and monoclonal antibodies, play a critical role in managing NMOSD by reducing the frequency and severity of relapses, ultimately improving patient outcomes.

The pathophysiological mechanisms of NMOSD involve complex inflammatory processes, with interleukin‐6 (IL‐6) serving as a key disease biomarker. Markedly elevated IL‐6 levels in cerebrospinal fluid and serum have been observed in NMOSD patients, particularly during relapses [6, 7]. This finding has led to the development of IL‐6 inhibitors as a therapeutic strategy for NMOSD [8]. Satralizumab, a humanized monoclonal antibody that targets the IL‐6 receptor, has shown promise in clinical trials [9, 10]. Two pivotal phase 3, randomized, double‐blind, and placebo‐controlled clinical trials, SAkuraSky and SAkuraStar, demonstrated that satralizumab significantly reduced relapse risk while maintaining a favorable tolerability profile. However, the sample sizes in these trials were relatively small (83 and 95 patients, respectively), potentially limiting their generalizability [9, 10]. Additionally, the phase 3 studies primarily assess efficacy and safety under controlled conditions, which may not fully reflect real‐world patient populations. To address these limitations, our study aims to evaluate the long‐term efficacy and safety of satralizumab in a larger and more diverse real‐world NMOSD cohort.

## 2 Methods

### 2.1 Study Design

The study used data from the TriNetX Research Network, a global federated health research network that provides real‐time access to de‐identified electronic health records. As of 2025, the TriNetX Global Network includes more than 170 million patients contributed by over 150 healthcare organizations across 17 countries. TriNetX employs a federated architecture, strict governance, and harmonization procedures to ensure privacy, data integrity, and international comparability. Its methodology and applications have been described in detail and validated across diverse therapeutic areas, and the platform has demonstrated notable utility in bridging the gap between randomized controlled trials and real‐world applications, particularly in rare diseases and heterogeneous patient populations [11, 12]. The study design adheres to the Strengthening the Reporting of Observational Studies in Epidemiology reporting (STROBE) guidelines [13] and complies with the principles of the Declaration of Helsinki [14]. This study was approved by the Joint Institutional Review Board of Taipei Medical University (reference number: N202502019), with a waiver of informed consent, as all data were deidentified and anonymous for analysis.

### 2.2 Study Population

We accessed data from the TriNetX Research Network on April 30, 2025, through the Taipei Medical University site (Taipei, Taiwan) to conduct the primary analysis. The primary dataset was extracted on April 30, 2025, and an additional query was conducted on September 25, 2025, to support sensitivity analyses. The diagnosis of NMOSD was solely made by selecting ICD codes in the TriNetX Research Network. To identify incident cases of NMOSD, we applied the International Statistical Classification of Diseases and Related Health Problems, Tenth Revision (ICD‐10) diagnostic code G36.0. For the 12‐month follow‐up cohort, eligible patients were identified between January 1, 2018, and April 30, 2024, ensuring at least 12 months of follow‐up after diagnosis. Patients with a minimum follow‐up of 24 or 36 months were similarly included in their respective cohorts. The 24‐month cohort included patients followed from January 1, 2018, to January 31, 2023, and the 36‐month cohort included those followed from January 1, 2018, to January 31, 2022. The index event for each cohort was defined as the initiation of immune therapy—either satralizumab or conventional immunosuppressants—marking the index date as the first recorded administration of the respective treatment.

### 2.3 Exposure and Control Group

The exposure group comprised NMOSD patients who received satralizumab monoclonal antibody, either as monotherapy or in combination with conventional immunosuppressants. Patients with prior treatment with other monoclonal antibodies, including eculizumab, inebilizumab, or tocilizumab, were excluded. The control group included NMOSD patients who did not receive any aforementioned monoclonal antibody treatment but were treated with conventional immunosuppressants, defined as oral prednisone, azathioprine, or mycophenolate mofetil, with or without rituximab. Patients with MOG‐IgG positivity were excluded from both groups due to a different disease entity [15]. Table S1 lists the codes of the ICD‐10 and medications applied for the inclusion and exclusion process.

### 2.4 Covariates

Covariates were assessed within a predefined time window, spanning from 3 years to 1 day before the index event. Variables were selected based on their potential to confound the association between immunosuppressive treatment and clinical outcomes. Demographic characteristics included age, gender, and race. Relevant comorbidities encompassed prior optic neuritis and transverse myelitis, as well as conditions such as hypertension, diabetes mellitus, Sjögren syndrome. Medication used during the predefined time window included oral prednisone, rituximab, azathioprine, and mycophenolate mofetil.

### 2.5 Main Outcomes

The efficacy outcome was the relapse rate at 1, 3, 6, 9, 12, 24, and 36 months following the index date. Relapse was defined as a composite outcome identified by the use of at least one of the following pharmacological interventions during the designated follow‐up period: methylprednisolone, intravenous infusion of human immune serum globulin (IVIg), therapeutic plasma exchange, plasmapheresis, therapeutic plasmapheresis using plasma as the primary replacement fluid, or single‐pass plasmapheresis.

Safety outcomes encompassed the incidence of infectious complications (sepsis, respiratory infection, urinary tract infection) and hematologic and mortality outcomes (anemia, neutropenia, and death), assessed at 1, 3, 6, 9, 12, 24, and 36 months following the index date. By monitoring these adverse events over time, we aimed to provide a comprehensive evaluation of the safety profile of NMOSD treatments.

### 2.6 Statistical Analysis

All statistical analyses were conducted using the built‐in statistical tools of the TriNetX network. Propensity score matching (PSM) was employed to balance baseline characteristics, which included age, sex, race, and the previously mentioned covariates between the satralizumab and conventional immunosuppressant groups. Propensity scores, representing the probability of being assigned to the satralizumab or conventional immunosuppressive agent groups, were generated using a logistic regression model incorporating these baseline variables. Matching was conducted at a 1:1 ratio using the nearest‐neighbor method with a caliper of 0.1. Standardized mean difference (SMD) was used to assess group balance, with an SMD < 0.1 indicating adequate balance. For smaller samples (*n* < 100), an SMD < 0.25 was considered acceptable due to increased variability [16, 17]. Additionally, *p* values from Student's *t* test or the chi‐square test were reported to support balance assessment in these smaller samples. Kaplan–Meier survival curves were used to estimate the crude incident rates, and log‐rank tests were performed to compare differences between the satralizumab and conventional immunosuppressant groups. The risk ratios (RRs) and risk differences (RDs) were respectively calculated from ratios and differences of cumulative incidence between the satralizumab and the conventional immunosuppressant groups. The number needed to treat (NNT) was obtained from the inverse of the RDs. A *p* value of < 0.05 was considered statistically significant. In the TriNetX platform, event counts ≤ 10 were not reported directly but instead displayed as “10” or shown as percentages, according to TriNetX governance policies protecting patient privacy.

### 2.7 Sensitivity Analysis

Several sensitivity analyses were conducted to evaluate the robustness of the findings. First, we examined efficacy outcomes among patients with documented AQP4‐IgG positivity, given its role as the key biomarker and treatment indication for satralizumab. This analysis was designed to confirm the robustness of the main results in a subgroup with complete laboratory data.

Second, to address treatment background, we compared satralizumab monotherapy with conventional immunosuppressants, excluding the small number of patients who received satralizumab in combination with other agents. Third, given that rituximab is widely used off‐label as a standard therapy in NMOSD, we included it in the conventional immunosuppressant group for the primary analysis but also conducted a direct comparison of satralizumab versus rituximab alone [18, 19, 20].

## 3 Results

### 3.1 Participants Characteristics

**FIGURE 1:**
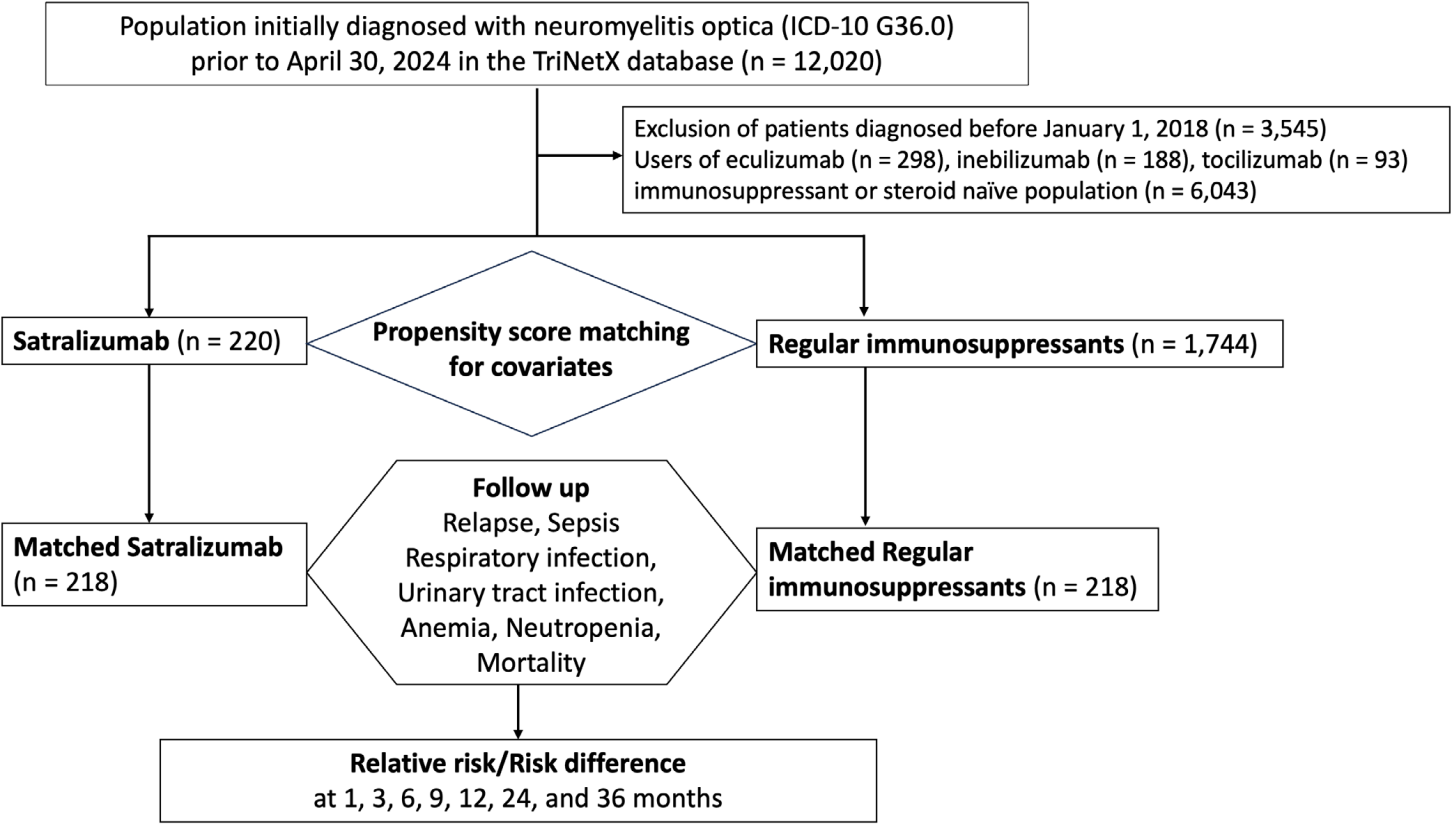
The study flow diagram.

Figure 1 illustrates the enrollment process of study participants. A total of 220 patients were included in the satralizumab group, while 1744 patients met the criteria for the conventional immunosuppressant control group. Table 1 presents the baseline characteristics and underlying conditions before and after PSM. Following PSM, 218 patients remained in each group. The majority of participants were female (approximately 86% in both groups), with a median age of 49.8 and 48.5 years in the satralizumab and conventional groups, respectively. The racial distribution in the satralizumab group was 42.7% White, 35.8% African American, closely mirroring that of the control group. Approximately one‐third of the patients in both groups had a clinical history of optic neuritis (33.9% in satralizumab and 35.8% in the conventional immunosuppressant group). Hypertension (25.7% in satralizumab vs. 24.3% in control) and diabetes mellitus (13.3% in satralizumab vs. 10.1% in control) were the most prevalent comorbidities. After PSM, almost all covariates achieved balance, with SMD < 0.1.

**TABLE 1:** Participants characteristics.

	Before propensity score matching (*N* = 1964)		SMD	After propensity score matching (*N* = 436)		SMD
	Satralizumab	Conventional immunosuppressants		Satralizumab	Conventional immunosuppressants	
Number of patients	220	1744		218	218	
Age at index, years, mean (SD)	49.8 (17.0)	50.5 (19.0)	0.039	49.8 (17.0)	48.5 (18.4)	0.071
Female, *N* (%)	193 (87.7)	1319 (75.6)	0.317*	191 (87.6)	187 (85.8)	0.054
Race, *N* (%)						
White	94 (42.7)	736 (42.2)	0.011	93 (42.7)	104 (47.7)	0.102*
Black or African American	79 (35.9)	390 (22.4)	0.301*	78 (35.8)	71 (32.6)	0.068
Comorbidities, *N* (%)						
Previous optic neuritis attack	74 (33.6)	596 (34.2)	0.011	74 (33.9)	77 (35.8)	0.029
Hypertension	57 (25.9)	456 (26.1)	0.005	56 (25.7)	53 (24.3)	0.032
Diabetes mellitus	30 (13.6)	221 (12.7)	0.029	29 (13.3)	22 (10.1)	0.100
Sjögren syndrome	10 (4.5)	114 (6.5)	0.087	10 (4.6)	10 (4.6)	< 0.001
Previous myelitis	42 (19.1)	299 (17.1)	0.051	40 (18.3)	38 (17.4)	0.024
Medication use, *N* (%)						
Oral prednisone	120 (54.5)	672 (38.5)	0.325*	118 (54.1)	126 (57.8)	0.074
Rituximab	76 (34.5)	254 (14.6)	0.477*	74 (33.9)	77 (34.9)	0.029
Azathioprine	10 (4.5)	245 (14.0)	0.332*	10 (4.6)	10 (4.6)	< 0.001
Mycophenolate mofetil	34 (15.5)	392 (22.5)	0.180*	34 (15.6)	33 (15.1)	0.013

*Note*: Categorical variables were expressed as number (%).

Abbreviations: SD, standard deviation; SMD, Standardized mean difference.

* Statistically significant difference was defined as SMD > 0.1. This may impact results, particularly for small cohorts and infrequent outcomes.

### 3.2 Efficacy Outcome

**FIGURE 2:**
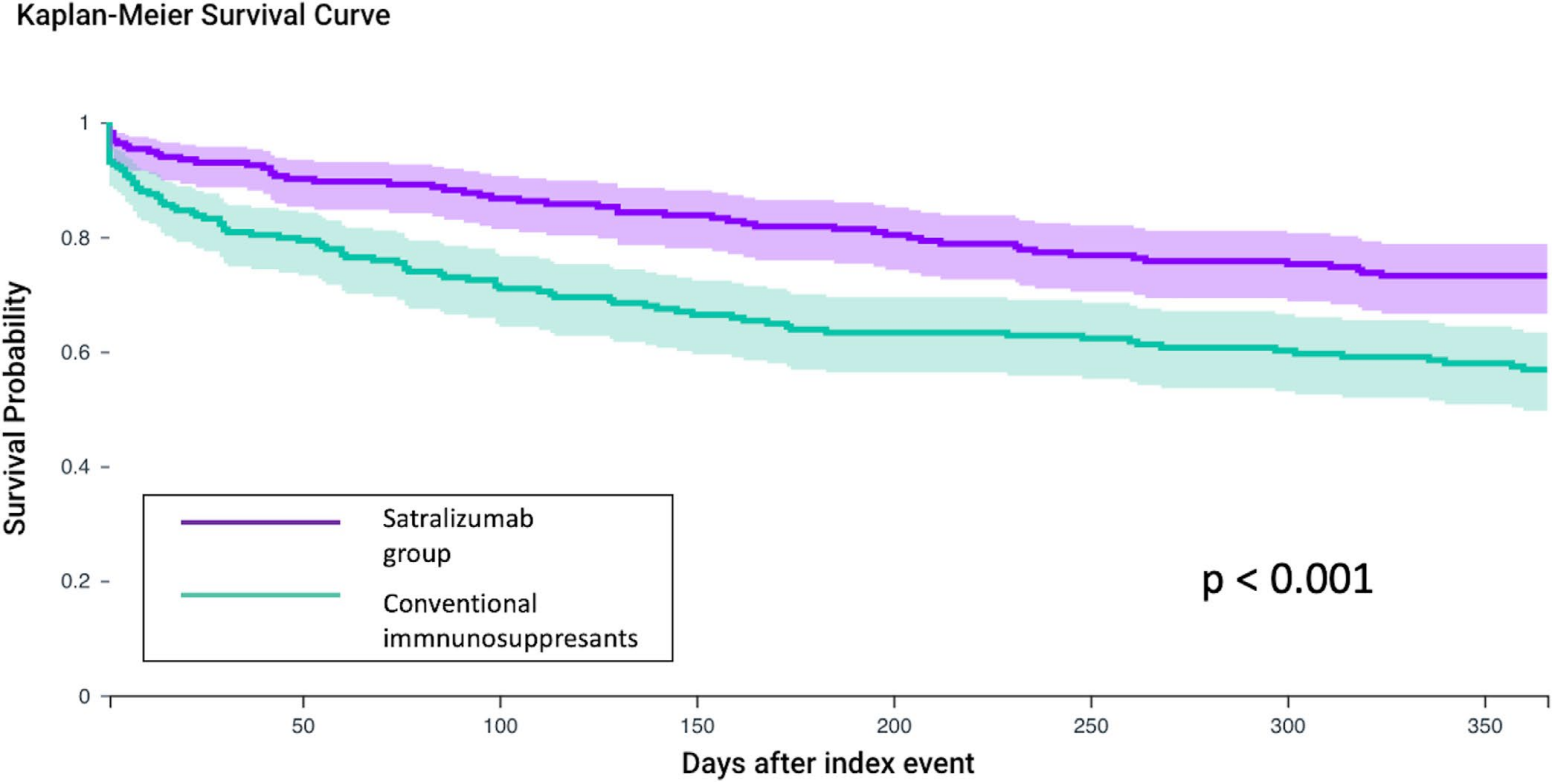
Effect of satralizumab on the relapse rates. Satralizumab exhibited a reduced relapse rate over 12 months in the matched neuromyelitis optica spectrum disorder cohort (218 patient pairs).

Table 2 summarizes relapse rates comparing satralizumab with conventional immunosuppressants. Follow‐up was available for 218 matched pairs at 12 months, 118 pairs at 24 months, and 54 pairs at 36 months, reflecting progressively smaller subsets of the same matched cohort due to follow‐up maturity. Patients treated with satralizumab experienced significantly lower relapse rates across all follow‐up intervals, with RRs consistently favoring satralizumab: 1 month (RR: 0.38, 95% CI 0.21–0.66), 3 months (RR: 0.43, 95% CI 0.28–0.66), 6 months (RR: 0.50, 95% CI 0.33–0.70), 9 months (RR: 0.62, 95% CI 0.46–0.83), 12 months (RR: 0.63, 95% CI 0.47–0.83), 24 months (RR: 0.60, 95% CI 0.42–0.86) and 36 months (RR: 0.52, 95% CI 0.32–0.83). The Kaplan–Meier curve showed satralizumab was associated with a reduced relapse risk within the 1‐year follow‐up period (Figure 2). Across all follow‐up intervals, NNTs were consistently between 4 and 9.

**TABLE 2:** Efficacy outcomes based on follow‐up intervals.

Follow‐up intervals	Relapse, no. (%)		RD, % (95% CI)	RR (95% CI)	NNT	*p*
	Satralizumab	Conventional immunosuppressants				
1‐month follow‐up						
Number of patients	218	218				
Relapse	15 (6.9)	40 (18.3)	−11.5 (−17.6, −5.3)	0.38 (0.21, 0.66)	9	< 0.001*
3‐month follow‐up						
Number of patients	218	218				
Relapse	25 (11.5)	58 (26.6)	−15.1 (−22.4, −7.9)	0.43 (0.28, 0.66)	7	< 0.001*
6‐month follow‐up						
Number of patients	218	218				
Relapse	38 (17.4)	76 (34.9)	−17.4 (−25.5, −9.3)	0.50 (0.33, 0.70)	6	< 0.001*
9‐month follow‐up						
Number of patients	218	218				
Relapse	50 (22.9)	81 (37.2)	−14.2 (−22.7, −5.7)	0.62 (0.46, 0.83)	7	0.001*
12‐month follow‐up						
Number of patients	218	218				
Relapse	55 (25.2)	88 (40.4)	−15.1 (−23.8, −6.4)	0.63 (0.47, 0.83)	7	0.002*
24‐month follow‐up						
Number of patients	118	118				
Relapse	32 (27.1)	53 (44.9)	−17.8 (−29.8, −5.8)	0.60 (0.42, 0.86)	6	0.005*
36‐month follow‐up						
Number of patients	54	54				
Relapse	16 (29.6)	31 (57.4)	−27.8 (−45.7, −9.8)	0.52 (0.32, 0.83)	4	0.007*

*Note: p* values were calculated for relative risk (RR). Sample sizes at 12, 24, and 36 months reflect progressively smaller subsets of the same matched cohort, determined by follow‐up maturity at the fixed data cutoff (April 30, 2025). Patients enrolled through 2024 contribute to 12‐month follow‐up, those enrolled through 2023 to 24‐month follow‐up, and those enrolled through 2022 to 36‐month follow‐up.

Abbreviations: CI, confidence interval; NNT, number needed to treat; RD, risk difference; RR, relative risk.

* Statistical significance (*p* < 0.05).

### 3.3 Safety Outcomes

Table 3 presents infectious complications, including sepsis, respiratory tract infection, and urinary tract infection (UTI). Table 4 summarizes hematologic and mortality outcomes, including anemia, neutropenia, and death. Across all follow‐up intervals, there were no statistically significant differences between the satralizumab and conventional immunosuppressant groups. Notably, UTI incidence appeared numerically higher in the satralizumab group at 24 and 36 months, although this did not reach statistical significance. Overall, these findings suggest a comparable safety profile between the two treatment approaches.

**TABLE 3:** Safety outcomes: infectious events (sepsis, respiratory tract infection, urinary tract infection).

Follow‐up intervals	Outcome, *N* (%)		RD, % (95% CI)	RR (95% CI)	NNT	*p*
	Satralizumab	Conventional immunosuppressants				
1‐month, *N*	218	218				
Sepsis	< 10^a^ (< 4.6)	< 10^a^ (< 4.6)	0 (−3.9, 3.9)	1.00 (0.43, 2.35)	—	1.000
RTI	0	0	NA	NA	—	NA
UTI	< 10^a^ (< 4.6)	15 (6.9)	−2.3 (−6.7, 2.1)	0.67 (0.31, 1.45)	−44	0.303
3‐month, *N*	218	218				
Sepsis	< 10^a^ (< 4.6)	< 10^a^ (< 4.6)	0 (−3.9, 3.9)	1.00 (0.43, 2.35)	—	1.000
RTI	0	0	NA	NA	—	NA
UTI	13 (6.0)	21 (9.6)	−3.7 (−8.7, 1.4)	0.62 (0.32, 1.21)	−28	0.153
6‐month, *N*	218	218				
Sepsis	< 10^a^ (< 4.6)	< 10^a^ (< 4.6)	0 (−3.9, 3.9)	1.00 (0.43, 2.35)	—	1.000
RTI	< 10^a^ (< 4.6)	0	4.6 (1.8, 7.4)	NA	22	NA
UTI	20 (9.2)	24 (11.0)	−1.8 (−7.5, 3.8)	0.83 (0.48, 1.46)	−56	0.525
9‐month, *N*	218	218				
Sepsis	< 10^a^ (< 4.6)	< 10^a^ (< 4.6)	0 (−3.9, 3.9)	1.00 (0.43, 2.35)	—	1.000
RTI	< 10^a^ (< 4.6)	0	4.6 (1.8, 7.4)	NA	22	NA
UTI	26 (11.9)	31 (14.2)	−2.3 (−8.6, 4.0)	0.84 (0.52, 1.36)	−44	0.478
12‐month, *N*	218	218				
Sepsis	< 10^a^ (< 4.6)	< 10^a^ (< 4.6)	0 (−3.9, 3.9)	1.00 (0.43, 2.35)	—	1.000
RTI	< 10^a^ (< 4.6)	0	4.6 (1.8, 7.4)	NA	22	NA
UTI	29 (13.3)	33 (15.1)	−1.8 (−8.4, 4.7)	0.89 (0.55, 1.40)	−56	0.583
24‐month, *N*	118	118				
Sepsis	< 10^a^ (< 8.5)	< 10^a^ (< 8.5)	0 (−7.1, 7.1)	1.00 (0.43, 2.31)	—	1.000
RTI	< 10^a^ (< 8.5)	0	8.5 (3.4, 13.5)	NA	12	NA
UTI	33 (28.0)	22 (18.6)	9.3 (−1.4, 20.0)	1.50 (0.93, 2.41)	11	0.094
36‐month, *N*	54	54				
Sepsis	< 10^a^ (< 18.5)	< 10^a^ (< 18.5)	0 (−14.7, 14.7)	1.00 (0.45, 2.21)	—	1.000
RTI	< 10^a^ (< 18.5)	< 10^a^ (< 18.5)	0 (−14.7, 14.7)	1.00 (0.45, 2.21)	—	1.000
UTI	19 (35.2)	14 (25.9)	9.3 (−8.0, 26.5)	1.38 (0.76, 2.42)	11	0.276

*Note: p* value was calculated for risk difference (RD). Sample sizes at 12, 24, and 36 months reflect progressively smaller subsets of the same matched cohort, determined by follow‐up maturity at the fixed data cutoff (April 30, 2025). Patients enrolled through 2024 contribute to 12‐month follow‐up, those enrolled through 2023 to 24‐month follow‐up, and those enrolled through 2022 to 36‐month follow‐up.

Abbreviations: CI, confidence interval; *N*, number; NA, not applicable; NNT, number needed to treat; RD, risk difference; RR, relative risk; RTI, respiratory tract infection; UTI, urinary tract infection.

^a^ To protect patient privacy for small cohorts and infrequent outcomes in the TriNetX platform, numbers less than and equal to 10 are rounded up to 10.

**TABLE 4:** Safety outcomes: hematologic and mortality events (anemia, neutropenia, death).

Follow‐up intervals	Outcome, no. (%)		RD, % (95% CI)	RR (95% CI)	NNT	*p*
	Satralizumab	Conventional immunosuppressants				
1‐month, *N*	218	218				
Anemia	< 10^a^ (< 4.6)	< 10^a^ (< 4.6)	0 (−3.9, 3.9)	1 (0.43, 2.35)	—	1.000
Neutropenia	< 10^a^ (< 4.6)	< 10^a^ (< 4.6)	0 (−3.9, 3.9)	1 (0.43, 2.35)	—	1.000
Death	0	< 10^a^ (< 4.6)	−4.6 (−7.4, −1.8)	NA	−22	NA
3‐month, *N*	218	218				
Anemia	11 (5.0)	16 (5.0)	0 (−4.1, 4.1)	1 (0.44, 2.26)	—	1.000
Neutropenia	< 10^a^ (< 4.6)	< 10^a^ (< 4.6)	0 (−3.9, 3.9)	1 (0.43, 2.35)	—	1.000
Death	0	< 10^a^ (< 4.6)	−4.6 (−7.4, −1.8)	NA	−22	NA
6‐month, *N*	218	218				
Anemia	18 (8.3)	17 (7.8)	0.5 (−4.6, 5.6)	1.06 (0.56, 2.00)	200	0.860
Neutropenia	< 10^a^ (< 4.6)	< 10^a^ (< 4.6)	0 (−3.9, 3.9)	1 (0.43, 2.35)	—	1.000
Death	< 10^a^ (< 4.6)	< 10^a^ (< 4.6)	0 (−3.9, 3.9)	1 (0.43, 2.35)	—	1.000
9‐month, *N*	218	218				
Anemia	21 (9.6)	23 (10.6)	−0.9 (−6.6, 4.7)	0.91 (0.52, 1.60)	−112	0.750
Neutropenia	< 10^a^ (< 4.6)	< 10^a^ (< 4.6)	0 (−3.9, 3.9)	1 (0.43, 2.35)	—	1.000
Death	< 10^a^ (< 4.6)	< 10^a^ (< 4.6)	0 (−3.9, 3.9)	1 (0.43, 2.35)	—	1.000
12‐month, *N*	218	218				
Anemia	24 (11.0)	26 (11.9)	−0.9 (−6.9, 5.1)	0.92 (0.55, 1.56)	−112	0.764
Neutropenia	< 10^a^ (< 4.6)	< 10^a^ (< 4.6)	0 (−3.9, 3.9)	1 (0.43, 2.35)	—	1.000
Death	< 10^a^ (< 4.6)	< 10^a^ (< 4.6)	0 (−3.9, 3.9)	1 (0.43, 2.35)	—	1.000
24‐month, *N*	118	118				
Anemia	22 (18.6)	16 (13.6)	5.1 (−4.3, 14.4)	1.38 (0.76, 2.48)	20	0.284
Neutropenia	< 10^a^ (< 8.5)	< 10^a^ (< 8.5)	0 (−7.1, 7.1)	1 (0.43, 2.35)	—	1.000
Death	< 10^a^ (< 8.5)	< 10^a^ (< 8.5)	0 (−7.1, 7.1)	1 (0.43, 2.35)	—	1.000
36‐month, *N*	54	54				
Anemia	13 (24.1)	12 (22.2)	1.9 (−14.1, 17.8)	1.08 (0.54, 2.16)	53	0.828
Neutropenia	< 10^a^ (< 18.5)	< 10^a^ (< 18.5)	0 (−14.7, 14.7)	1 (0.45, 2.21)	—	1.000
Death	< 10^a^ (< 18.5)	< 10^a^ (< 18.5)	0 (−14.7, 14.7)	1 (0.45, 2.21)	—	1.000

*Note: p* value was calculated for risk difference (RD). Sample sizes at 12, 24, and 36 months reflect progressively smaller subsets of the same matched cohort, determined by follow‐up maturity at the fixed data cutoff (April 30, 2025). Patients enrolled through 2024 contribute to 12‐month follow‐up, those enrolled through 2023 to 24‐month follow‐up, and those enrolled through 2022 to 36‐month follow‐up.

Abbreviations: CI, confidence interval; *N*, number; NA, not applicable; NNT, number needed to treat; RD, risk difference; RR, relative risk.

^a^ To protect patient privacy for small cohorts and infrequent outcomes in the TriNetX platform, numbers less than and equal to 10 are rounded up to 10.

### 3.4 Sensitivity Analysis: Documented AQP4‐IgG Positivity

After PSM, baseline characteristics were balanced between groups (Table S2). Satralizumab was associated with lower relapse rates compared with conventional immunosuppressants at 1, 3, 6, 9, and 12 months (RRs 0.50–0.54; NNTs 5–8; Table S3), consistent with the primary analysis. The Kaplan–Meier curve showed satralizumab was associated with a reduced relapse risk over 12 months in patients with documented AQP4‐IgG positivity (Figure S1). Longer follow‐up (24–36 months) was not feasible because relapse events in this subgroup fell below TriNetX's minimum reporting threshold (≤ 10).

### 3.5 Sensitivity Analysis: Patients With Satralizumab Monotherapy

After PSM, baseline characteristics between the satralizumab monotherapy group and the conventional immunosuppressant group were well balanced (Table S4). Relapse outcomes across follow‐up intervals were consistent with the primary analysis, including at 6 months (16.5% vs. 33.0%; RR, 0.50; 95% CI, 0.34–0.73), 12 months (22.2% vs. 40.2%; RR, 0.55; 95% CI, 0.40–0.76), 24 months (27.1% vs. 44.9%; RR, 0.49; 95% CI, 0.33–0.73), and 36 months (25.0% vs. 50.0%; RR, 0.50; 95% CI, 0.28–0.88) (Table S5). The Kaplan–Meier curve showed satralizumab monotherapy was associated with a reduced relapse risk over 12 months (Figure S2).

### 3.6 Sensitivity Analysis: Satralizumab Monotherapy vs. Rituximab Monotherapy

We conducted a direct comparison between satralizumab monotherapy and rituximab monotherapy. After PSM, the two groups were well balanced (Table S6). Satralizumab monotherapy was consistently associated with lower relapse rates across follow‐up intervals, including 6 months (16.5% vs. 55.2%; RR, 0.30; 95% CI, 0.21–0.42), 12 months (22.2% vs. 70.1%; RR, 0.32; 95% CI, 0.24–0.42), 24 months (26.3% vs. 69.5%; RR, 0.38; 95% CI, 0.26–0.54), and 36 months (22.2% vs. 73.3%; RR, 0.30; 95% CI, 0.17–0.54) (Table S7). Kaplan–Meier analysis similarly demonstrated a reduced relapse risk with satralizumab monotherapy compared to rituximab during the 12‐month follow‐up (Figure S3).

## 4 Discussion

This multicenter retrospective cohort study supports the findings of previous clinical trials, demonstrating that satralizumab significantly reduces relapse rates in NMOSD patients compared to conventional immunosuppressants, without increasing the incidence of adverse events, in overall and AQP4‐IgG‐seropositive patients. The NNTs remained relatively small between 4 and 9 across 36 months of follow‐up.

### 4.1 The Underlying Pathophysiologic Mechanism of NMOSD

The pathophysiologic mechanism underlying NMOSD is primarily driven by AQP4‐IgG, which plays a key role in mediating central nervous system injury [21]. AQP4 is a water channel protein crucial for maintaining astrocyte homeostasis, and is copiously expressed in astrocyte foot processes in the central nervous system, particularly within the brain, spinal cord, and optic nerve [22]. AQP4‐IgG, a circulating immunoglobulin G1 antibody, is found in approximately 70% of NMOSD patients but is absent in multiple sclerosis and other neurological diseases [23, 24]. The binding of AQP4‐IgG to AQP4 initiates a cascade of pathological events, including activation of the classical complement pathway, infiltration of granulocyte and macrophage, and subsequent damage to the oligodendrocyte. This inflammatory response ultimately leads to neuronal injury and cell death, contributing to the severe neurological deficits observed in NMOSD [25].

### 4.2 Implications of IL‐6 Targeting

Recent real‐world evidence from Japan has shown that biologics, including IL‐6 receptor inhibitors, achieve substantially greater relapse reduction than conventional immunosuppressants without compromising safety [26]. IL‐6 plays a critical role in modulating the humoral immune response and T‐cell differentiation pathways. Initially identified as stimulatory factor‐2 for B cells, IL‐6 promotes the differentiation of activated B cells into antibody‐producing cells [27]. Furthermore, IL‐6, in combination with transforming growth factor beta 1 (TGF‐β1) and IL‐23, promotes the differentiation of naïve T cells into Th17 cells [28], which are implicated in autoimmune inflammation. Beyond its immunomodulatory functions, IL‐6 contributes to the pathogenesis of NMOSD by disrupting the integrity of the blood–brain barrier, allowing AQP4‐IgG antibodies to infiltrate the central nervous system, leading to astrocyte damage [8]. By targeting IL‐6 receptors, satralizumab effectively mitigates this inflammatory cascade, thereby reducing disease activity and preventing relapse in NMOSD.

In this context, the marked treatment effect observed as early as 1 month in our study is noteworthy. This may reflect satralizumab's loading regimen (Weeks 0, 2, and 4), which rapidly achieves therapeutic concentrations and enables early IL‐6 receptor blockade [10]. Patients initiating satralizumab may also have had higher baseline inflammatory activity, making relapse reduction more visible in the early phase compared with those maintained on conventional immunosuppressants. Prior randomized trials similarly reported early stabilization of disease activity after treatment initiation [10]. Taken together, these factors likely explain the early reduction in relapse risk observed in our real‐world analysis.

### 4.3 Time Window Selection

Determining our time window of covariates retrieval, spanning from 3 years to 1 day before the index events, was balanced between statistical power and variability. A wider time window encompasses more events, thereby gaining more statistical power. On the other hand, a narrower time window decreases the variability from time‐dependent factors such as different definitions of the comorbidities or different clinical practices and reduces noise by having more immediate outcomes following the time of the covariates' retrieval [12].

### 4.4 Sensitivity Analysis

We conducted several sensitivity analyses, including stratification by AQP4‐IgG serostatus, restriction to satralizumab monotherapy, and direct comparison with rituximab. Across all approaches, the results were consistent with the primary analysis, supporting the robustness of our findings.

Notably, our primary analysis—satralizumab with or without concomitant conventional immunosuppressants—parallels the SAkuraSky trial, which evaluated satralizumab as an add‐on to background immunosuppressive therapy. In contrast, our sensitivity analysis of satralizumab monotherapy reflects the design of SAkuraStar, demonstrating that satralizumab remains effective even in the absence of concomitant agents. Together, these complementary perspectives strengthen confidence that the observed benefit is attributable to IL‐6 receptor blockade rather than background therapy alone.

Both pivotal clinical trials, SAkuraSky and SAkuraStar [9, 10], demonstrated that satralizumab's benefit in reducing relapse rates was more pronounced in AQP4‐IgG‐seropositive patients, whereas the effect was not significant among seronegative individuals. Similarly, our sensitivity analysis (Table S3) showed consistent relapse reduction in the AQP4‐IgG–seropositive patients, supporting this biologically plausible treatment‐response pattern. Larger sample sizes will be required to confirm efficacy in seronegative patients.

Our findings align with the pooled analysis of SAkuraSky and SAkuraStar, which reported a 58% reduction in protocol‐defined relapse (hazard ratio, 0.42; 95% CI, 0.25–0.71) in satralizumab‐treated patients compared to placebo [29]. Similarly, our study observed a 48% reduction in relapse rates at 36 months compared to conventional immunosuppressants. Notably, when restricted to the AQP4‐IgG seropositive group, the pooled analysis revealed an even greater reduction in relapse risk, reaching 75% (hazard ratio, 0.25; 95% CI, 0.12 to 0.50) [29].

Finally, rituximab is frequently used off‐label as a first‐line therapy for NMOSD and thus provides an important clinical benchmark. Satralizumab monotherapy demonstrated consistently lower relapse rates compared with rituximab monotherapy across follow‐up intervals of up to 36 months. This direct comparison extends trial evidence by contrasting two biologic strategies in a real‐world cohort and suggests that IL‐6 receptor blockade may provide superior relapse prevention, supporting satralizumab as a viable alternative in clinical practice.

### 4.5 Safety and Tolerability

Infections remain the most common adverse events with satralizumab trials, particularly upper respiratory tract infections [30]. In our study, infection and hematologic event rates did not differ significantly from those with conventional immunosuppressants, consistent with pivotal SAkuraSky and SAkuraStar trials. The SAkuraSky trial similarly found no differences in leukopenia and anemia incidence between satralizumab and placebo (leukopenia: 14.6% vs. 9.5%; anemia: 7.3% vs. 11.9%) [10]. Long‐term extension data from SAkuraMoon, an open‐label study following patients from SAkuraSky and SAkuraStar, confirmed a stable safety profile with Satralizumab over nearly 7 years of exposure, with low rates of serious infections and no new safety concerns [31].

Post‐marketing surveillance in Japan and US claims analyses likewise showed lower infection and sepsis rates in satralizumab‐treated patients compared with broader NMOSD populations [31, 32]. In line with these reports, our study observed no significant difference in adverse events between the satralizumab and conventional immunosuppressant groups during 3 years of follow‐up. With its favorable safety profiles, satralizumab has also demonstrated strong treatment adherence.

### 4.6 Limitations and Strengths

Our study has several limitations. First, relapse was defined using surrogate markers such as receipt of high‐dose steroids, IVIg, or plasmapheresis, which may not perfectly capture clinical disease activity. These interventions can be used for conditions other than NMOSD relapse, while milder relapses managed without such treatments may have been missed. Nonetheless, this approach has been applied in prior real‐world studies [33].

Second, the TriNetX platform records medication prescriptions but does not capture dose, duration, or cumulative volume. This limitation has not been acknowledged in TriNetX‐based studies, including real‐world multiple sclerosis research [34]. Because of this, we could not directly assess the potential modifying effect of background glucocorticoid or immunosuppressant use. To mitigate this limitation, we performed a sensitivity analysis restricted to satralizumab monotherapy versus conventional immunosuppressants, which yielded consistent results and supports that the observed effect is attributable to satralizumab itself.

Third, although we compared satralizumab monotherapy with rituximab monotherapy, the number of patients receiving satralizumab in combination with other immunosuppressants was too small for reliable subgroup analysis. Finally, in the TriNetX platform, event counts ≤ 10 were not reported directly but instead displayed as “10” or shown as percentages, according to TriNetX governance policies protecting patient privacy. While this reduces granularity for rare outcomes, it does not affect the overall interpretation: satralizumab remained consistently more effective in reducing relapse compared with both conventional immunosuppressants and rituximab, and demonstrated comparable safety to conventional immunosuppressants. As with any retrospective analysis, residual confounding and potential misclassification due to coding errors cannot be completely excluded. However, sensitivity analyses across multiple sensitivity analyses strengthen the validity of our findings.

Our study also has notable strengths. Unlike prior placebo‐controlled trials, we used an active comparator—conventional immunosuppressants—providing a clinically relevant benchmark for real‐world practice. The use of a large, international, multicenter dataset enhances generalizability. Finally, by restricting analyses to patients treated without other IL‐6‐targeted agents, we ensured that observed effects could be attributed specifically to satralizumab. Importantly, our findings complement emerging real‐world data from Japan, where interim multicenter chart reviews also confirmed high relapse‐free rates with satralizumab in AQP4‐IgG–positive patients [35].

## 5 Conclusion

Compared to conventional immunosuppressants, satralizumab was more effective in preventing relapse and demonstrated a favorable safety profile in treating NMOSD patients. Future research is needed to validate these outcomes by including more diverse populations.

## Author Contributions

S.‐F.L., L.‐T.L., and H.‐A.L. contributed to the conception and design of the study. S.‐F.L. and H.‐A.L. were responsible for the acquisition and analysis of data. S.‐F.L. and L.‐T.L. contributed to drafting the manuscript. All authors reviewed and approved the final version of the manuscript and take full responsibility for its content.

## Conflicts of Interest

The authors declare no conflicts of interest.

## Supporting information


**Figure S1:** Effect of satralizumab in patients with documented AQP4‐IgG positivity. Satralizumab was associated with reduced relapse rates over 12 months in the AQP4‐IgG–positive subgroup (78 patient pairs).
**Figure S2:** Effect of satralizumab monotherapy. Satralizumab monotherapy was associated with reduced relapse rates over 12 months compared with conventional immunosuppressants (194 patient pairs).
**Figure S3:** Effect of satralizumab monotherapy compared with rituximab monotherapy. Satralizumab monotherapy was associated with reduced relapse rates over 12 months compared with rituximab monotherapy (194 patient pairs).

**Table S1:** Codes for defining diagnoses, medications, and procedures.
**Table S2:** Participants Characteristics with Positive AQP‐4.
**Table S3:** Efficacy Outcomes based on follow‐up intervals for Participants with positive AQP‐4.
**Table S4:** Baseline characteristics of patients treated with satralizumab monotherapy versus conventional immunosuppressants.
**Table S5:** Efficacy outcomes for satralizumab monotherapy versus conventional immunosuppressants across follow‐up intervals.
**Table S6:** Baseline characteristics of patients treated with satralizumab versus rituximab.
**Table S7:** Efficacy outcomes for satralizumab versus rituximab across follow‐up intervals.

## Data Availability

This study used de‐identified patient‐level data from the TriNetX Research Network, which aggregates electronic health records from participating healthcare organizations under established data use agreements. Because access to TriNetX is contractually restricted, the raw datasets analyzed in this study cannot be publicly shared. Researchers interested in using TriNetX data may apply for access directly through TriNetX (https://trinetx.com).

## References

[1] R. A. Kessler , M. A. Mealy , and M. Levy , “Treatment of Neuromyelitis Optica Spectrum Disorder: Acute, Preventive, and Symptomatic,” Current Treatment Options in Neurology 18, no. 1 (2015): 2. 10.1007/s11940-015-0387-9 PMC4807395 26705758

[2] S. Huda , D. Whittam , M. Bhojak , et al., “Neuromyelitis Optica Spectrum Disorders,” Clinical Medicine 19, no. 2 (2019): 169–176. 30872305 10.7861/clinmedicine.19-2-169 PMC6454358

[3] J. Brody , M. A. Hellmann , R. Marignier , I. Lotan , and H. Stiebel‐Kalish , “Neuromyelitis Optica Spectrum Disorder: Disease Course and Long‐Term Visual Outcome,” Journal of Neuro‐Ophthalmology 36, no. 4 (2016): 356–362. 27348750 10.1097/WNO.0000000000000403

[4] P. Cabre , A. González‐Quevedo , M. Bonnan , et al., “Relapsing Neuromyelitis Optica: Long Term History and Clinical Predictors of Death,” Journal of Neurology, Neurosurgery, and Psychiatry 80, no. 10 (2009): 1162–1164. 19762908 10.1136/jnnp.2007.143529

[5] A. Berthele , M. Levy , D. M. Wingerchuk , et al., “A Single Relapse Induces Worsening of Disability and Health‐Related Quality of Life in Patients With Neuromyelitis Optica Spectrum Disorder,” Frontiers in Neurology 14 (2023): 1099376. 37114235 10.3389/fneur.2023.1099376 PMC10126826

[6] P. O. Barros , T. Cassano , J. Hygino , et al., “Prediction of Disease Severity in Neuromyelitis Optica by the Levels of Interleukin (IL)‐6 Produced During Remission Phase,” Clinical and Experimental Immunology 183, no. 3 (2016): 480–489. 26472479 10.1111/cei.12733 PMC4750605

[7] A. Uzawa , M. Mori , K. Arai , et al., “Cytokine and Chemokine Profiles in Neuromyelitis Optica: Significance of Interleukin‐6,” Multiple Sclerosis Journal 16, no. 12 (2010): 1443–1452. 20739337 10.1177/1352458510379247

[8] K. Fujihara , J. L. Bennett , J. de Seze , et al., “Interleukin‐6 in Neuromyelitis Optica Spectrum Disorder Pathophysiology,” Neurology Neuroimmunology & Neuroinflammation 7, no. 5 (2020): e841. 32820020 10.1212/NXI.0000000000000841 PMC7455314

[9] A. Traboulsee , B. M. Greenberg , J. L. Bennett , et al., “Safety and Efficacy of Satralizumab Monotherapy in Neuromyelitis Optica Spectrum Disorder: A Randomised, Double‐Blind, Multicentre, Placebo‐Controlled Phase 3 Trial,” Lancet Neurology 19, no. 5 (2020): 402–412. 32333898 10.1016/S1474-4422(20)30078-8 PMC7935419

[10] T. Yamamura , I. Kleiter , K. Fujihara , et al., “Trial of Satralizumab in Neuromyelitis Optica Spectrum Disorder,” New England Journal of Medicine 381, no. 22 (2019): 2114–2124. 31774956 10.1056/NEJMoa1901747

[11] M. B. Palchuk , J. W. London , D. Perez‐Rey , et al., “A Global Federated Real‐World Data and Analytics Platform for Research,” JAMIA Open 6, no. 2 (2023): ooad035. 37193038 10.1093/jamiaopen/ooad035 PMC10182857

[12] R. J. Ludwig , M. Anson , H. Zirpel , et al., “A Comprehensive Review of Methodologies and Application to Use the Real‐World Data and Analytics Platform TriNetX,” Frontiers in Pharmacology 16 (2025): 1516126. 40129946 10.3389/fphar.2025.1516126 PMC11931024

[13] E. von Elm , D. G. Altman , M. Egger , S. J. Pocock , P. C. Gøtzsche , and J. P. Vandenbroucke , “The Strengthening the Reporting of Observational Studies in Epidemiology (STROBE) Statement: Guidelines for Reporting Observational Studies,” Journal of Clinical Epidemiology 61, no. 4 (2008): 344–349. 18313558 10.1016/j.jclinepi.2007.11.008

[14] World Medical Association , “Declaration of Helsinki: Ethical Principles for Medical Research Involving Human Subjects Revised October 7, 2000,” HIV Clinical Trials 2, no. 1 (2001): 92–95. 11590516 10.1310/GTFR-2DRX-M6YE-ELXR

[15] S. I. Taha , S. I. Bakr , N. T. Fouad , D. Zamzam , and Y. A. Mohamed , “Clinical Characteristics of Anti‐Myelin Oligodendrocyte Glycoprotein Antibody Among Aquaporin‐4 Negative Neuromyelitis Optica Spectrum Disorders in Egyptian Patients,” Scientific Reports 15, no. 1 (2025): 1438. 39789036 10.1038/s41598-024-83760-2 PMC11718070

[16] E. A. Stuart , “Matching Methods for Causal Inference: A Review and a Look Forward,” Statistical Science 25, no. 1 (2010): 1–21. 20871802 10.1214/09-STS313 PMC2943670

[17] P. C. Austin and E. A. Stuart , “Moving Towards Best Practice When Using Inverse Probability of Treatment Weighting (IPTW) Using the Propensity Score to Estimate Causal Treatment Effects in Observational Studies,” Statistics in Medicine 34, no. 28 (2015): 3661–3679. 26238958 10.1002/sim.6607 PMC4626409

[18] S.‐H. Kim , J.‐H. Min , S.‐M. Kim , et al., “Evaluating Rituximab Failure Rates in Neuromyelitis Optica Spectrum Disorder: A Nationwide Real‐World Study From South Korea,” Journal of Clinical Neurology 21, no. 2 (2025): 131–136. 40065454 10.3988/jcn.2024.0485 PMC11896746

[19] T. Ongphichetmetha , J. Jitprapaikulsan , S. Siritho , et al., “Efficacy and Safety of Rituximab in Multiple Sclerosis and Neuromyelitis Optica Spectrum Disorder,” Scientific Reports 14, no. 1 (2024): 3503. 38347079 10.1038/s41598-024-53838-y PMC10861443

[20] S. Demuth and N. Collongues , “Disease‐Modifying Treatments for Neuromyelitis Optica Spectrum Disorder in the Context of a New Generation of Biotherapies,” Revue Neurologique (Paris) 181, no. 1 (2025): 42–51. 10.1016/j.neurol.2024.01.008 38553270

[21] M. C. Kowarik , M. Dzieciatkowska , S. Wemlinger , et al., “The Cerebrospinal Fluid Immunoglobulin Transcriptome and Proteome in Neuromyelitis Optica Reveals Central Nervous System‐Specific B Cell Populations,” Journal of Neuroinflammation 12, no. 1 (2015): 19. 25626447 10.1186/s12974-015-0240-9 PMC4323273

[22] J. E. Rash , T. Yasumura , C. S. Hudson , P. Agre , and S. Nielsen , “Direct Immunogold Labeling of Aquaporin‐4 in Square Arrays of Astrocyte and Ependymocyte Plasma Membranes in Rat Brain and Spinal Cord,” Proceedings of the National Academy of Sciences 95, no. 20 (1998): 11981–11986. 10.1073/pnas.95.20.11981 PMC21751 9751776

[23] A. Zekeridou and V. A. Lennon , “Aquaporin‐4 Autoimmunity,” Neurology Neuroimmunology & Neuroinflammation 2, no. 4 (2015): e110. 26185772 10.1212/NXI.0000000000000110 PMC4442096

[24] D. K. Sato , D. Callegaro , F. M. de Haidar Jorge , et al., “Cerebrospinal Fluid Aquaporin‐4 Antibody Levels in Neuromyelitis Optica Attacks,” Annals of Neurology 76, no. 2 (2014): 305–309. 24977390 10.1002/ana.24208 PMC4173125

[25] M. C. Papadopoulos , J. L. Bennett , and A. S. Verkman , “Treatment of Neuromyelitis Optica: State‐of‐The‐Art and Emerging Therapies,” Nature Reviews. Neurology 10, no. 9 (2014): 493–506. 25112508 10.1038/nrneurol.2014.141 PMC4229040

[26] N. Yamazaki , T. Misu , Y. Matsumoto , et al., “The Real‐World Impact of Biologics for NMOSD: A Retrospective Single‐Center Study Compared With Natural Course and Conventional Treatments in Japanese,” Multiple Sclerosis and Related Disorders 92 (2024): 106176, https://pubmed.ncbi.nlm.nih.gov/39579645/. 39579645 10.1016/j.msard.2024.106176

[27] T. Tanaka and T. Kishimoto , “Targeting Interleukin‐6: All the Way to Treat Autoimmune and Inflammatory Diseases,” International Journal of Biological Sciences 8, no. 9 (2012): 1227–1236. 23136551 10.7150/ijbs.4666 PMC3491446

[28] G. R. D. Passos , D. K. Sato , J. Becker , and K. Fujihara , “Th17 Cells Pathways in Multiple Sclerosis and Neuromyelitis Optica Spectrum Disorders: Pathophysiological and Therapeutic Implications,” Mediators of Inflammation 2016, no. 1 (2016): 5314541. 26941483 10.1155/2016/5314541 PMC4749822

[29] B. R. Meher , R. R. Mohanty , A. Dash , et al., “Review of Satralizumab for Neuromyelitis Optica Spectrum Disorder: A New Biologic Agent Targeting the Interleukin‐6 Receptor,” Cureus 16, no. 2 (2024): e55100, https://www.cureus.com/articles/227543‐review‐of‐satralizumab‐for‐neuromyelitis‐optica‐spectrum‐disorder‐a‐new‐biologic‐agent‐targeting‐the‐interleukin‐6‐receptor. 38558672 10.7759/cureus.55100 PMC10978816

[30] S. Fung and M. Shirley , “Satralizumab: A Review in Neuromyelitis Optica Spectrum Disorder,” CNS Drugs 37, no. 4 (2023): 363–370. 36933107 10.1007/s40263-023-00995-9

[31] B. M. Greenberg , K. Fujihara , B. Weinshenker , et al., “Analysis of Infection Rates in Neuromyelitis Optica Spectrum Disorder: Comparing Satralizumab Treatment in SAkuraMoon, Post‐Marketing, and US‐Based Health Claims Data,” Multiple Sclerosis and Related Disorders 99 (2025): 106444, https://pubmed.ncbi.nlm.nih.gov/40288333/. 40288333 10.1016/j.msard.2025.106444

[32] T. Yamamura , N. Isobe , I. Kawachi , et al., “Safety and Effectiveness of Satralizumab in Japanese Patients With Neuromyelitis Optica Spectrum Disorder: A 6‐Month Interim Analysis of Post‐Marketing Surveillance,” Neurology and Therapy 13, no. 5 (2024): 1361–1383. 39012406 10.1007/s40120-024-00640-7 PMC11393251

[33] J. L. Hsu , M.‐Y. Cheng , J. J. Su , et al., “Impact of Comorbidities on Relapsing Rates of Neuromyelitis Optica Spectrum Disorders: Insights From a Longitudinal Study in Taiwan,” Multiple Sclerosis and Related Disorders 87 (2024): 105683. 38761695 10.1016/j.msard.2024.105683

[34] J. R. Earla , G. J. Hutton , J. D. Thornton , and R. R. Aparasu , “Factors Associated With Prescribing Oral Disease Modifying Agents in Multiple Sclerosis: A Real‐World Analysis of Electronic Medical Records,” Multiple Sclerosis and Related Disorders 45 (2020): 102334. 32629400 10.1016/j.msard.2020.102334

[35] K. Fujihara , N. Isobe , K. Miyamoto , et al., “Effectiveness of Satralizumab in a Real‐World Clinical Setting in Japan: Interleukin‐6 Receptor Inhibition in Neuromyelitis Optica Spectrum Disorder: A Six‐Month Interim Analysis of a Multicenter Medical Chart Review,” Multiple Sclerosis and Related Disorders 98 (2025): 106384. 40203604 10.1016/j.msard.2025.106384

