# Supplementary material for: Real‐World Investigation of Satralizumab in Patients With Neuromyelitis Optica Spectrum Disease
Source: Ann Clin Transl Neurol. 2025 Nov 14;13(4):665–75. doi: 10.1002/acn3.70246 (PMC13071087; doi:10.1002/acn3.70246)
Supplement: Supplementary file 1 — Figure S1: Effect of satralizumab in patients with documented AQP4‐IgG positivity. Satralizumab was associated with reduced relapse rates over 12 months in the AQP4‐IgG–positive subgroup (78 patient pairs). Figure S2: Effect of satralizumab monotherapy. Satralizumab monotherapy was associated with reduced relapse rates over 12 months compared with conventional immunosuppressants (194 patient pairs). Figure S3: Effect of satralizumab monotherapy compared with rituximab monotherapy. Satralizumab monotherapy was associated with reduced relapse rates over 12 months compared with rituximab monotherapy (194 patient pairs). [file ACN3-13-665-s001.docx]

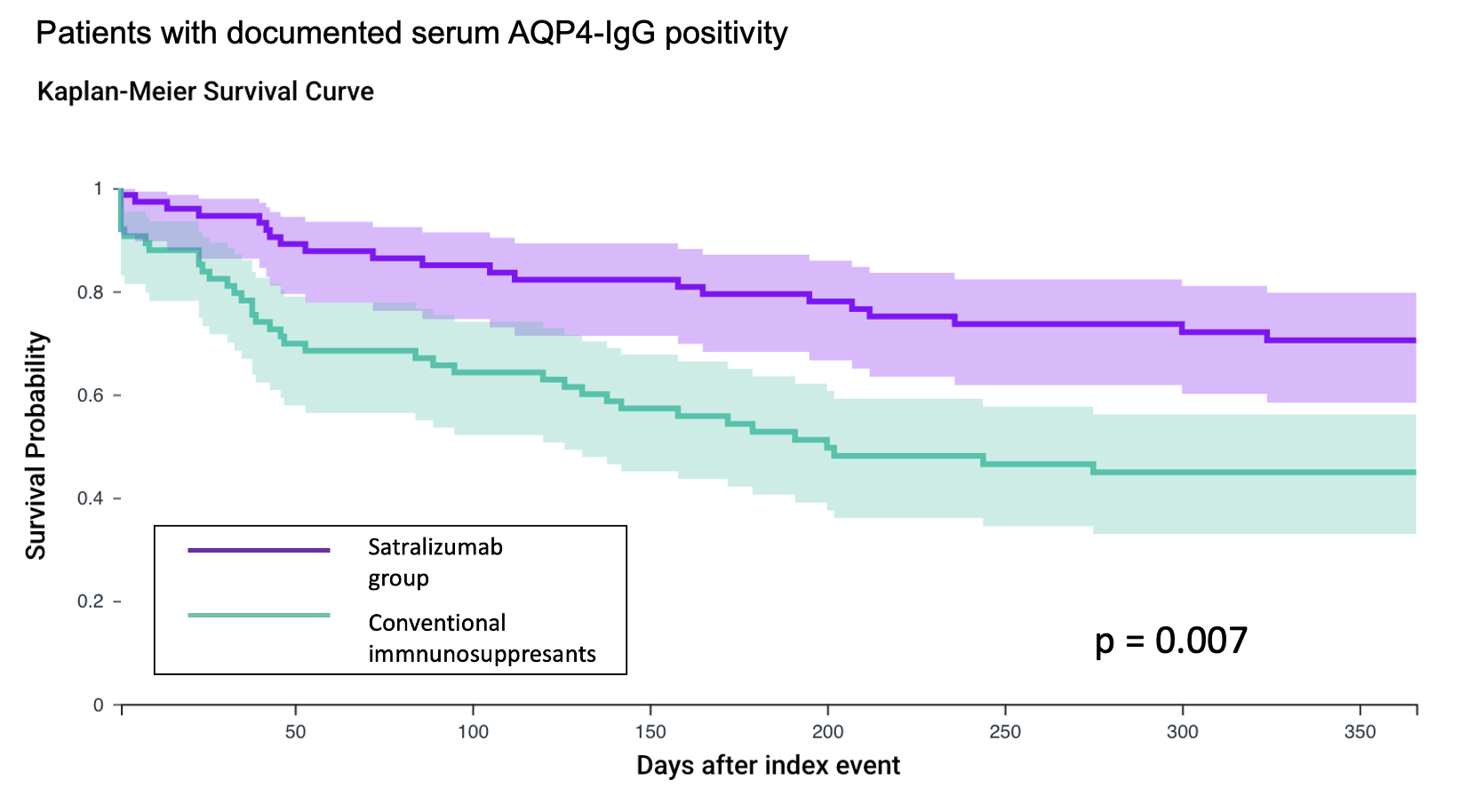


**Supplemental Figure 1. Effect of satralizumab in patients with documented AQP4-IgG positivity.** Satralizumab was associated with reduced relapse rates over 12 months in the AQP4-IgG–positive subgroup (78 patient pairs).


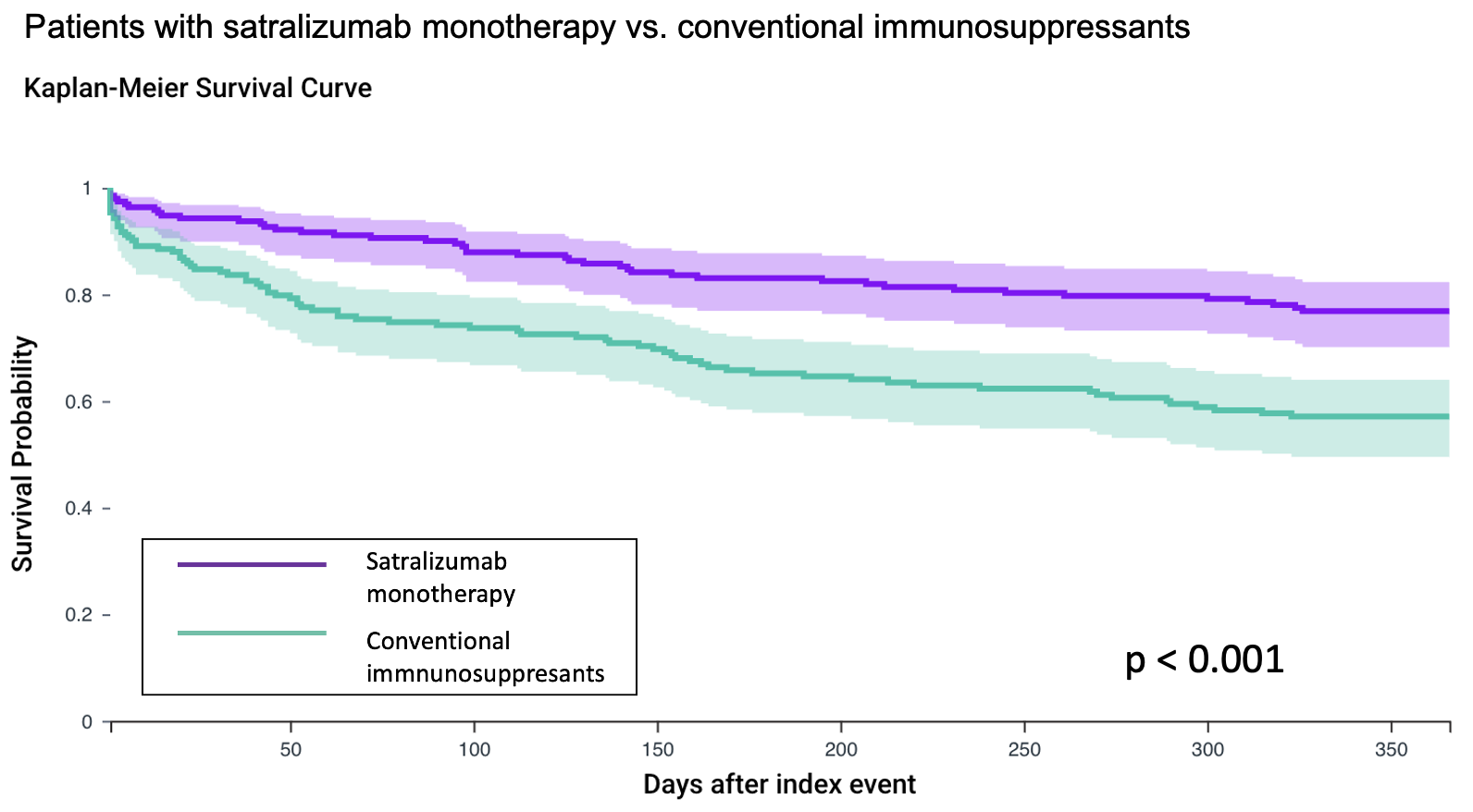


**Supplemental Figure 2. Effect of satralizumab monotherapy.** Satralizumab monotherapy was associated with reduced relapse rates over 12 months compared with conventional immunosuppressants (194 patient pairs).

**
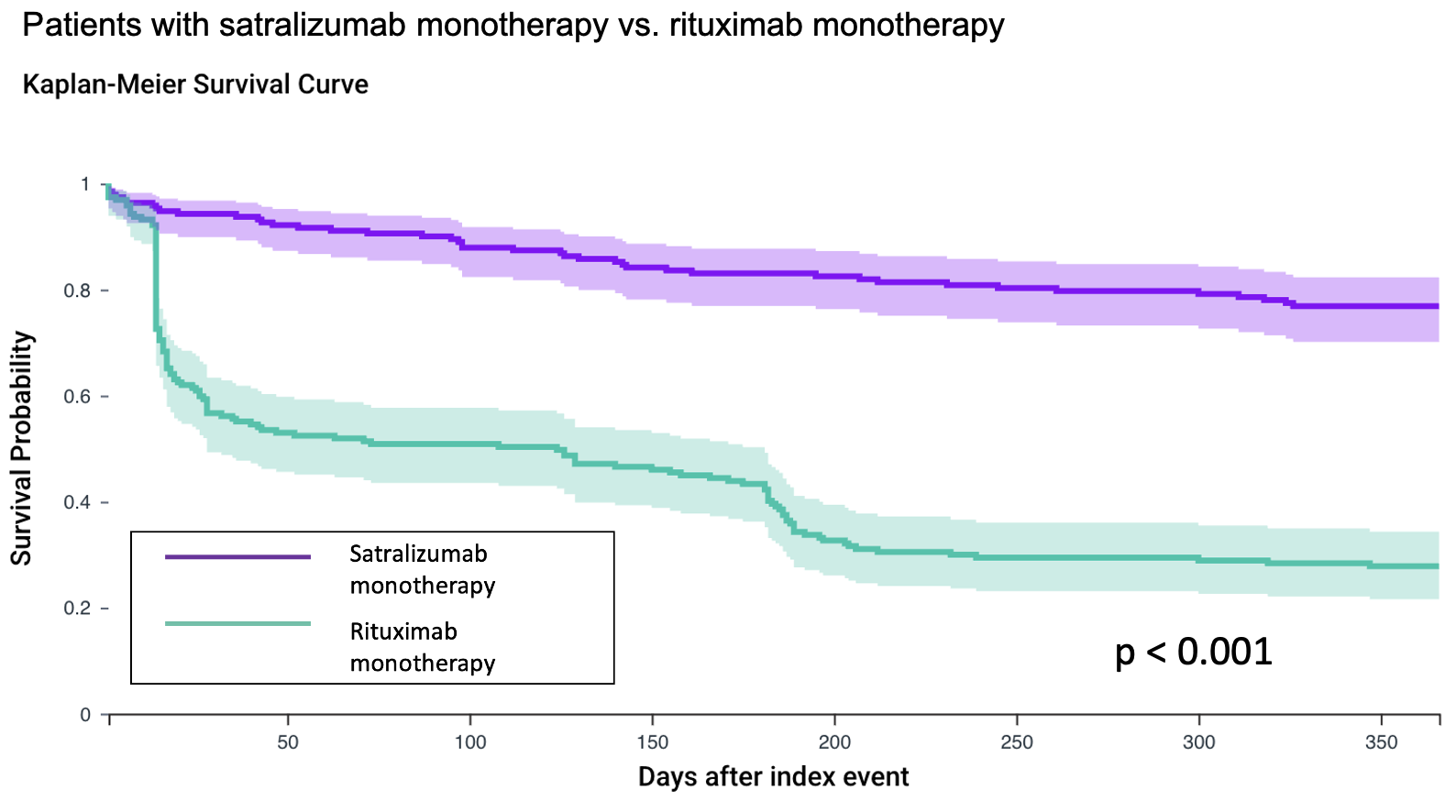
**

**Supplemental Figure 3. Effect of satralizumab monotherapy compared with rituximab monotherapy.** Satralizumab monotherapy was associated with reduced relapse rates over 12 months compared with rituximab monotherapy (194 patient pairs).
