# Supplementary material for: Real‐World Investigation of Satralizumab in Patients With Neuromyelitis Optica Spectrum Disease
Source: Ann Clin Transl Neurol. 2025 Nov 14;13(4):665–75. doi: 10.1002/acn3.70246 (PMC13071087; doi:10.1002/acn3.70246)
Supplement: Supplementary file 2 — Table S1: Codes for defining diagnoses, medications, and procedures. Table S2: Participants Characteristics with Positive AQP‐4. Table S3: Efficacy Outcomes based on follow‐up intervals for Participants with positive AQP‐4. Table S4: Baseline characteristics of patients treated with satralizumab monotherapy versus conventional immunosuppressants. Table S5: Efficacy outcomes for satralizumab monotherapy versus conventional immunosuppressants across follow‐up intervals. Table S6: Baseline characteristics of patients treated with satralizumab versus rituximab. Table S7: Efficacy outcomes for satralizumab versus rituximab across follow‐up intervals. [file ACN3-13-665-s002.docx]

**Supplemental Materials.**

**Supplemental Table 1.** Codes for defining diagnoses, medications, and procedures.

**Supplemental Table 2.** Participants Characteristics with Positive AQP-4.

**Supplemental Table 3.** Efficacy Outcomes based on follow-up intervals for Participants with positive AQP-4.

**Supplemental Table 4.** Baseline characteristics of patients treated with satralizumab monotherapy versus conventional immunosuppressants.

**Supplemental Table 5.** Efficacy outcomes for satralizumab monotherapy versus conventional immunosuppressants across follow-up intervals.

**Supplemental Table 6.** Baseline characteristics of patients treated with satralizumab versus rituximab

**Supplemental Table 7.** Efficacy outcomes for satralizumab versus rituximab across follow-up intervals.

Supplemental Table 1. Codes for defining diagnoses, medications, and procedures

| *Diagnosis* | *ICD-10 code* |
| --- | --- |
| Neuromyelitis optica | G36 |
| Hypertension | I10-I1A |
| Diabetes mellitus | E08-E13 |
| Optic neuritis | H46 |
| Acute transverse myelitis in demyelinating disease of central nervous system | G37.3 |
| Sjögren syndrome | M35.0 |
| *Medication* | *RxNorm code* |
| satralizumab | 2391541 |
| eculizumab | 591781 |
| inebilizumab | 2373951 |
| tocilizumab | 612865 |
| prednisone | 8640 |
| prednisolone | 8638 |
| mycophenolic acid | 7145 |
| mycophenolate | 265323 |
| mycophenolate mofetil | 68149 |
| azathioprine | 1256 |
| methylprednisolone | 6902 |
| *Lab results* | *Code* |
| Serum AQP-4 IgG positivity | 43638-6 |
| Serum MOG IgG positivity | 46718-3 |
| *Procedures* | *SNOMED or ICD-10-PCS* |
| Intravenous infusion of human immune serum globulin (IVIg) | 116805008 |
| Therapeutic plasma exchange | 225067007 |
| Plasmapheresis | 20720000 |
| Therapeutic plasmapheresis using plasma as the major replacement fluid | 19647005 |
| Pheresis of Plasma, Single | 6A550Z3 |

ICD-10-PCS, ICD-10-Procedure Coding System; SNOMED, Systematized Nomenclature of Medicine

Supplemental Table 2. Participants Characteristics with Positive AQP-4

|  | Before propensity score matching  (N= 674) | | SMD | After propensity score matching  (N= 156) | | SMD | *P* value |
| --- | --- | --- | --- | --- | --- | --- | --- |
|  | Satralizumab | Conventional immunosuppressants |  | Satralizumab | Conventional immunosuppressants |  |  |
| Number of patients | 78 | 596 |  | 78 | 78 |  |  |
| Age at index, years, mean (SD) | 52.1 (16.7) | 52.3 (17.2) | 0.011 | 52.1 (16.7) | 54.0 (17.3) | 0.110 | 0.493 |
| Female, N (%) | 67 (85.9) | 488 (81.9) | 0.109 | 67 (85.9) | 69 (88.5) | 0.077 | 0.632 |
| Race, N (%) |  |  |  |  |  |  |  |
| White | 32 (41.0) | 261 (43.8) | 0.056 | 32 (41.0) | 36 (46.2) | 0.104 | 0.518 |
| Black or African American | 31 (39.7) | 161 (27.0) | 0.272* | 31 (39.7) | 20 (25.6) | 0.304* | 0.060 |
| Comorbidities, N (%) |  |  |  |  |  |  |  |
| Previous optic neuritis attack | 37 (47.4) | 195 (32.7) | 0.304* | 37 (47.4) | 35 (44.9) | 0.051 | 0.748 |
| Diabetes mellitus | 12 (15.4) | 79 (13.3) | 0.061 | 12 (15.4) | 11 (14.1) | 0.036 | 0.821 |
| Sjögren syndrome | 10 (12.8) | 40 (6.7) | 0.207 | 10 (12.8) | 10 (12.8) | <0.001 | 1.000 |
| Previous myelitis | 20 (25.6) | 118 (19.8) | 0.140 | 20 (25.6) | 17 (21.8) | 0.091 | 0.572 |
| Medication use, N (%) |  |  |  |  |  |  |  |
| Oral prednisone | 47 (60.3) | 187 (31.4) | 0.606* | 47 (60.3) | 50 (64.1) | 0.079 | 0.620 |
| Rituximab | 31 (39.7) | 108 (18.1) | 0.491* | 31 (39.7) | 27 (34.6) | 0.106 | 0.508 |
| Azathioprine | 10 (12.8) | 45 (7.6) | 0.175 | 10 (12.8) | 10 (12.8) | <0.001 | 1.000 |
| Mycophenolate mofetil | 10 (12.8) | 51 (8.6) | 0.138 | 10 (12.8) | 10 (12.8) | <0.001 | 1.000 |

Abbreviations: AQP-4, aquaporin-4 antibody; IgG, immunoglobulin G; MOG, myelin oligodendrocyte glycoprotein antibody; SMD. Standardized mean difference; SD, standard deviation. Continuous variables were expressed as mean+/- standard deviation. Categorical variables were expressed as number (%).

*Statistically significant difference was defined as SMD > 0.25 for smaller samples.

Supplemental Table 3. Efficacy Outcomes based on follow-up intervals for Participants with positive AQP-4

| Follow-up intervals | Relapse, No. (%) | |  |  |  |  |
| --- | --- | --- | --- | --- | --- | --- |
|  | Satralizumab | Conventional Immunosuppressants | RD, % (95% CI) | RR (95% CI) | NNT | P value |
| 1-month follow-up |  |  |  |  |  |  |
| Number of patients | 78 | 78 |  |  |  |  |
| Relapse | 10 (12.8) | 20 (25.6) | -12.8 (-25.0, -0.6) | 0.50 (0.25, 0.99) | 8 | 0.042* |
| 3-month follow-up |  |  |  |  |  |  |
| Number of patients | 78 | 78 |  |  |  |  |
| Relapse | 11 (11.5) | 21 (26.6) | -12.8 (-25.3, -0.3) | 0.52 (0.27, 0.99) | 8 | 0.042* |
| 6-month follow-up |  |  |  |  |  |  |
| Number of patients | 78 | 78 |  |  |  |  |
| Relapse | 15 (19.2) | 30 (38.5) | -19.2 (-33.1, -5.3) | 0.50 (0.29, 0.85) | 6 | 0.008* |
| 9-month follow-up |  |  |  |  |  |  |
| Number of patients | 75 | 75 |  |  |  |  |
| Relapse | 19 (25.3) | 36 (48.0) | -22.7 (-37.7, -7.7) | 0.53 (0.34, 0.83) | 5 | 0.004* |
| 12-month follow-up |  |  |  |  |  |  |
| Number of patients | 75 | 75 |  |  |  |  |
| Relapse | 21 (28.0) | 39 (52.0) | -24.0 (-39.2, -8.8) | 0.54 (0.35, 0.82) | 6 | 0.003* |

RR, relative risk; RD, risk difference; NNT, number needed to treat; CI, confidence interval; P value were calculated for relative risk (RR). *Statistical significance (*P* < 0.05).

Supplemental Table 4. Participants Characteristics for Satralizumab monotherapy

|  | Before propensity score matching  (N= 2232) | | SMD | After propensity score matching  (N= 388) | | SMD |
| --- | --- | --- | --- | --- | --- | --- |
|  | Satralizumab monotherapy | Conventional immunosuppressants |  | Satralizumab monotherapy | Conventional immunosuppressants |  |
| Number of patients | 194 | 2038 |  | 194 | 194 |  |
| Age at index, years, mean (SD) | 50.4 (17.4) | 50.8 (18.7) | 0.022 | 50.4 (17.4) | 49.7 (18.7) | 0.036 |
| Female, N (%) | 167 (86.1) | 1547 (75.9) | 0.262* | 167 (86.1) | 165 (85.1) | 0.029 |
| Race, N (%) |  |  |  |  |  |  |
| White | 83 (42.8) | 834 (40.9) | 0.038 | 83 (42.8) | 88 (45.4) | 0.052 |
| Black or African American | 73 (37.6) | 475 (23.3) | 0.315* | 73 (37.6) | 68 (35.1) | 0.054 |
| Comorbidities, N (%) |  |  |  |  |  |  |
| Previous optic neuritis attack | 63 (32.5) | 603 (29.6) | 0.062 | 63 (32.5) | 75 (38.7) | 0.130 |
| Hypertension | 55 (28.4) | 480 (23.6) | 0.110* | 55 (28.4) | 50 (25.8) | 0.058 |
| Diabetes mellitus | 24 (12.4) | 227 (11.1) | 0.038 | 24 (12.4) | 22 (11.3) | 0.032 |
| Sjögren syndrome | 10 (5.2) | 115 (6.6) | 0.022 | 10 (5.2) | 11 (5.7) | 0.023 |
| Previous myelitis | 35 (18.0) | 310 (15.2) | 0.076 | 35 (18.0) | 30 (15.5) | 0.069 |

Abbreviations: SMD. Standardized mean difference; SD, standard deviation. Continuous variables were expressed as mean+/- standard deviation. Categorical variables were expressed as number (%).

*Statistically significant difference was defined as SMD > 0.1.

Supplemental Table 5. Efficacy outcomes for satralizumab monotherapy versus conventional immunosuppressants across follow-up intervals.

| Follow-up intervals | Relapse, No. (%) | |  |  |  |  |
| --- | --- | --- | --- | --- | --- | --- |
|  | Satralizumab monotherapy | Conventional Immunosuppressants | RD, % (95% CI) | RR (95% CI) | NNT | P value |
| 1-month follow-up |  |  |  |  |  |  |
| Number of patients | 194 | 194 |  |  |  |  |
| Relapse | 11 (5.7) | 29 (14.9) | -9.2 (-15.3, -3.3) | 0.38 (0.20, 0.74) | 11 | 0.003* |
| 3-month follow-up |  |  |  |  |  |  |
| Number of patients | 194 | 194 |  |  |  |  |
| Relapse | 19 (9.8) | 48 (24.7) | -14.9 (-22.3, -7.6) | 0.40 (0.24, 0.65) | 7 | <0.001* |
| 6-month follow-up |  |  |  |  |  |  |
| Number of patients | 194 | 194 |  |  |  |  |
| Relapse | 32 (16.5) | 64 (33.0) | -16.5 (-24.9, -8.1) | 0.50 (0.34, 0.73) | 6 | <0.001* |
| 9-month follow-up |  |  |  |  |  |  |
| Number of patients | 194 | 194 |  |  |  |  |
| Relapse | 38 (19.6) | 71 (36.6) | -17.0 (-25.8, -8.2) | 0.54 (0.38, 0.75) | 6 | <0.001* |
| 12-month follow-up |  |  |  |  |  |  |
| Number of patients | 194 | 194 |  |  |  |  |
| Relapse | 43 (22.2) | 78 (40.2) | -18.0 (-27.1, -9.0) | 0.55 (0.40, 0.76) | 6 | <0.001* |
| 24-month follow-up |  |  |  |  |  |  |
| Number of patients | 102 | 102 |  |  |  |  |
| Relapse | 25 (27.1) | 51 (44.9) | -25.5 (-38.3, -12.7) | 0.49 (0.33, 0.73) | 4 | <0.001* |
| 36-month follow-up |  |  |  |  |  |  |
| Number of patients | 48 | 48 |  |  |  |  |
| Relapse | 12 (25.0) | 24 (50.0) | -25.0 (-43.7, -6.3) | 0.50 (0.28, 0.88) | 4 | 0.011* |

RR, relative risk; RD, risk difference; NNT, number needed to treat; CI, confidence interval; P value were calculated for relative risk (RR). *Statistical significance (*P* < 0.05).

Supplemental Table 6. Baseline characteristics of patients treated with satralizumab versus rituximab

|  | Before propensity score matching  (N= 2150) | | SMD | After propensity score matching  (N= 388) | | SMD |
| --- | --- | --- | --- | --- | --- | --- |
|  | Satralizumab  monotherapy | Rituximab monotherapy |  | Satralizumab  monotherapy | Rituximab monotherapy |  |
| Number of patients | 194 | 1956 |  | 194 | 194 |  |
| Age at index, years, mean (SD) | 50.4 (17.4) | 49.4 (16.9) | 0.059 | 50.4 (17.4) | 48.9 (17.4) | 0.082 |
| Female, N (%) | 167 (86.1) | 1505 (76.9) | 0.237* | 167 (86.1) | 169 (87.1) | 0.030 |
| Race, N (%) |  |  |  |  |  |  |
| White | 83 (42.8) | 940 (48.1) | 0.106* | 83 (42.8) | 74 (38.1) | 0.095 |
| Black or African American | 73 (37.6) | 532 (27.2) | 0.224* | 73 (37.6) | 79 (40.7) | 0.063 |
| Comorbidities, N (%) |  |  |  |  |  |  |
| Previous optic neuritis attack | 63 (32.5) | 732 (37.4) | 0.104 | 63 (32.5) | 64 (33.0) | 0.011 |
| Hypertension | 55 (28.4) | 518 (26.5) | 0.042 | 55 (28.4) | 50 (25.8) | 0.058 |
| Diabetes mellitus | 24 (12.4) | 211 (10.8) | 0.050 | 24 (12.4) | 20 (10.3) | 0.065 |
| Sjögren syndrome | 10 (5.2) | 92 (4.7) | 0.021 | 10 (5.2) | 10 (5.2) | <0.001 |
| Previous myelitis | 35 (18.0) | 418 (21.4) | 0.084 | 35 (18.0) | 38 (19.6) | 0.040 |

Abbreviations: SMD. Standardized mean difference; SD, standard deviation.

Continuous variables were expressed as mean+/- standard deviation. Categorical variables were expressed as number (%).

*Statistically significant difference was defined as SMD > 0.1.

Supplemental Table 7. Efficacy outcomes for satralizumab versus rituximab across follow-up intervals.

| Follow-up intervals | Relapse, No. (%) | |  |  |  |  |
| --- | --- | --- | --- | --- | --- | --- |
|  | Satralizumab  Monotherapy | Rituximab  Monotherapy | RD, % (95% CI) | RR (95% CI) | NNT | P value |
| 1-month follow-up |  |  |  |  |  |  |
| Number of patients | 194 | 194 |  |  |  |  |
| Relapse | 11 (5.7) | 82 (42.3) | -36.6 (-44.3, -28.9) | 0.13 (0.07, 0.24) | 3 | <0.001* |
| 3-month follow-up |  |  |  |  |  |  |
| Number of patients | 194 | 194 |  |  |  |  |
| Relapse | 19 (9.8) | 93 (47.9) | -38.1 (-46.3, -30.0) | 0.20 (0.13, 0.32) | 3 | <0.001* |
| 6-month follow-up |  |  |  |  |  |  |
| Number of patients | 194 | 194 |  |  |  |  |
| Relapse | 32 (16.5) | 107 (55.2) | -38.7 (-47.4, -29.9) | 0.30 (0.21, 0.42) | 3 | <0.001* |
| 9-month follow-up |  |  |  |  |  |  |
| Number of patients | 194 | 194 |  |  |  |  |
| Relapse | 38 (19.6) | 133 (68.6) | -49.0 (-57.6, -40.4) | 0.29 (0.21, 0.39) | 3 | <0.001* |
| 12-month follow-up |  |  |  |  |  |  |
| Number of patients | 194 | 194 |  |  |  |  |
| Relapse | 43 (22.2) | 136 (70.1) | -47.9 (-56.6, -39.2) | 0.32 (0.24, 0.42) | 3 | <0.001* |
| 24-month follow-up |  |  |  |  |  |  |
| Number of patients | 95 | 95 |  |  |  |  |
| Relapse | 25 (26.3) | 66 (69.5) | -43.2 (-56.0, -30.3) | 0.38 (0.26, 0.54) | 3 | <0.001* |
| 36-month follow-up |  |  |  |  |  |  |
| Number of patients | 45 | 45 |  |  |  |  |
| Relapse | 10 (22.2) | 33 (73.3) | -51.1 (-68.8, -33.4) | 0.30 (0.17, 0.54) | 2 | <0.001* |

RR, relative risk; RD, risk difference; NNT, number needed to treat; CI, confidence interval; P value were calculated for relative risk (RR). *Statistical significance (*P* < 0.05).
